# Computational Model Predicts the Effects of Targeting Cellular Metabolism in Pancreatic Cancer

**DOI:** 10.3389/fphys.2017.00217

**Published:** 2017-04-12

**Authors:** Mahua Roy, Stacey D. Finley

**Affiliations:** ^1^Biomedical Engineering, University of Southern CaliforniaLos Angeles, CA, USA; ^2^Chemical Engineering, University of Southern CaliforniaLos Angeles, CA, USA

**Keywords:** metabolic modeling, systems biology, kinetic model, sensitivity analysis, parameter optimization

## Abstract

Reprogramming of energy metabolism is a hallmark of cancer that enables the cancer cells to meet the increased energetic requirements due to uncontrolled proliferation. One prominent example is pancreatic ductal adenocarcinoma, an aggressive form of cancer with an overall 5-year survival rate of 5%. The reprogramming mechanism in pancreatic cancer involves deregulated uptake of glucose and glutamine and other opportunistic modes of satisfying energetic demands in a hypoxic and nutrient-poor environment. In the current study, we apply systems biology approaches to enable a better understanding of the dynamics of the distinct metabolic alterations in KRAS-mediated pancreatic cancer, with the goal of impeding early cell proliferation by identifying the optimal metabolic enzymes to target. We have constructed a kinetic model of metabolism represented as a set of ordinary differential equations that describe time evolution of the metabolite concentrations in glycolysis, glutaminolysis, tricarboxylic acid cycle and the pentose phosphate pathway. The model is comprised of 46 metabolites and 53 reactions. The mathematical model is fit to published enzyme knockdown experimental data. We then applied the model to perform *in silico* enzyme modulations and evaluate the effects on cell proliferation. Our work identifies potential combinations of enzyme knockdown, metabolite inhibition, and extracellular conditions that impede cell proliferation. Excitingly, the model predicts novel targets that can be tested experimentally. Therefore, the model is a tool to predict the effects of inhibiting specific metabolic reactions within pancreatic cancer cells, which is difficult to measure experimentally, as well as test further hypotheses toward targeted therapies.

## 1. Introduction

Pancreatic ductal adenocarcinoma (PDAC) is a particularly aggressive and challenging form of cancer (Hidalgo, 2010; Oberstein and Olive, 2013; Siegel et al., 2013; Blum and Kloog, 2014) that is highly resistant to conventional chemotherapy. Mutations mediated by the KRAS or MYC oncogenes, found in 95% of cases of PDAC (Almoguera et al., 1988; Uemura et al., 2004; Löhr et al., 2005; Hezel et al., 2006; Kimmelman, 2015), promote reprogramming of the cellular metabolism, enabling the cancer cells to optimally use available resources (Ying et al., 2012). Specifically, KRAS promotes glucose uptake (Donahue and Dawson, 2016) and rewiring of glucose and glutamine metabolism (Kerr et al., 2016) to satisfy the excess demand for nutrients and cellular resources needed to sustain proliferation. The cells use glycolysis (glucose metabolism) to generate cellular resources needed to produce more cells. Similarly, increased glutamine consumption enables the cells to meet the larger demand for nitrogen needed to generate building blocks such as amino acids and lipids (Eagle, 1955; Vasseur et al., 2010; Pavlova and Thompson, 2016). The cells exhibit high survival and minimal death, even when the primary nutrients and energetic resources are scarce, suggesting that the cells adapt to the challenging conditions by altering their metabolism (Yoshida, 2015). This reprogramming of metabolic pathways is considered to be an emerging hallmark of most cancers (Hanahan and Weinberg, 2011) and is a driver of malignant growth. Moreover, the metabolic stress that occurs as a result of KRAS-mediated metabolic alterations can lead to further mutations and continued cell proliferation and tumor progression (Cairns et al., 2011; Misale et al., 2012). For these reasons, the dysregulated metabolic pathways can be used to identify biomarkers to support cancer diagnosis (Chung et al., 2003; Serkova and Boros, 2005; Pelicano et al., 2006). The altered metabolism also represents potential therapeutic targets (Macheda et al., 2005).

Pancreatic cancer cells are particularly reliant on glutamine to sustain proliferation and promote cell survival. Glutamine is a conditionally essential amino acid that fuels the tricarboxylic acid (TCA) cycle. Upon being taken up by the cell, glutamine is converted to glutamate by the glutaminase (GLS) enzyme, and then enters the TCA cycle as α-ketoglutarate (Wise and Thompson, 2010). Interestingly, PDAC has been characterized by non-canonical metabolism of glutamine, whereby the enzyme glutamic-oxaloacetic transaminase (GOT1) catalyzes the conversion of cytosolic aspartate to oxaloacetate. This enzyme is used in pancreatic cancer, instead of the glutamate dehydrogenase enzyme (GLUD1) used by normal cells to convert glutamate derived from glutamine to α-ketoglutarate in the mitochondria (McGivan and Chappell, 1975; Newsholme et al., 2003).

Glutamate, α-ketoglutarate, and aspartate are all important glutamine metabolism intermediates needed for cell proliferation. Glutamate-pyruvate transaminase (GPT), also known as alanine amino-transferase, transfers nitrogen from glutamate to pyruvate to make alanine and α-ketoglutarate. This nitrogen supports amino acid synthesis needed to produce cellular building blocks (i.e., lipids and nucleic acids). The α-ketoglutarate obtained by the conversion of glutamate promotes citrate production and lipid biosynthesis (Wise et al., 2011; Metallo et al., 2012). Aspartate is converted to oxaloacetate (Cohen et al., 2015), which is further converted to malate and then to pyruvate through the action of malic enzyme (ME1). The action of ME1 increases the NADPH/NADP ratio to maintain the redox balance and to replenish the glutathione (GSH) pool to quench the reactive oxygen species (ROS) (Gaglio et al., 2011). Given the importance of glutamine in pancreatic cancer, the enzymes that catalyze its metabolism, including GLS, GOT1, and ME1, are potential targets for impeding cell growth (Weinberg et al., 2010; Gross et al., 2014). For example, knocking down GOT1 activity alters the cells reductive capacity and is shown to inhibit cell proliferation *in vitro* and tumor growth *in vivo* (Son et al., 2013).

Pancreatic cancer cells also utilize the glycolytic pathway to metabolize glucose. Glycolysis converts glucose to pyruvate, most of which is used to form lactate, producing some ATP, rather than incorporated into the TCA cycle for ATP production. The increased reliance on glycolysis, despite the fact that oxidative phosphorylation is more efficient in generating ATP is termed the “*Warburg effect*” (Warburg, 1956) and has been widely studied (Vander Heiden et al., 2009). However, glycolysis enables the cells to meet their demand for precursors needed for biomass synthesis, which outweighs their energetic demands for ATP or NADH from the TCA cycle. The demand for the generation of amino acids, lipids, and nucleic acids is further satisfied by branching pathways that exploit the elevated glucose uptake, including the pentose phosphate pathway (PPP) (DeBerardinis et al., 2008; Weinberg et al., 2010; Patra and Hay, 2014). The PPP provides NADPH for macromolecule biosynthesis and quenching of reactive oxygen species (ROS), termed reductive biosynthesis. It also generates ribose-5-phosphate (R5P) required as a precursor for DNA and RNA biosynthesis (Recktenwald et al., 2008; DeNicola et al., 2011). Glucose metabolism has been targeted in attempts to inhibit cancer cell proliferation (El Mjiyad et al., 2011), and it remains a target in pancreatic cancer (Vander Heiden, 2011).

Mathematical modeling is necessary to understand metabolic reprogramming in cancer cells. Predictive mathematical models can incorporate the many metabolites, enzymes, and regulatory mechanisms that characterize cellular metabolism to enable a better understanding of the metabolic pathways (Vazquez et al., 2010; Alberghina et al., 2012; Cazzaniga et al., 2014; Le Novère, 2015). Many published metabolic modeling techniques have focused on constraint-based approaches in which certain physical, chemical, or biological constraints are applied to predict the metabolic phenotypes (Resendis-Antonio et al., 2010; Bordbar et al., 2014). These are steady state stoichiometric models that can predict the flux distributions, but fail to capture the kinetic aspects (time course of metabolite concentrations) in the system or time-varying heterogeneities that arise due to environmental fluctuations. When considering processes that are inherently transient, such as the effects of reprogramming of cancer metabolism, kinetic modeling is required to understand the dynamic relationship between metabolic fluxes and metabolite concentrations (Markert and Vazquez, 2015). Therefore, models that represent the metabolic pathways using a system of nonlinear ordinary differential equations (ODEs) are of particular importance. These kinetic models provide a mechanistic description of the transient dynamics of the system (Machado et al., 2012; Cazzaniga et al., 2014), as well as provide steady state measurements. When constructed and validated using experimental measurements, kinetic models can be used to perform *in silico* experiments to predict the dynamic effects of perturbing the metabolic network. In this way, the models are a valuable alternative to wet experiments that can be expensive and time-consuming.

In this study, we construct such a kinetic model of pancreatic cancer cell metabolism. Given the importance of glutamine and glucose metabolism in promoting pancreatic cancer cell proliferation, we apply the model to identify effective metabolic targets for impeding proliferation. The model is used to simulate the effects of altering specific metabolic enzymes and is a valuable tool to quantitatively understand the dynamics of cancer cell metabolism.

## 2. Materials and methods

### 2.1. Model structure and numerical implementation

**Figure 1:**
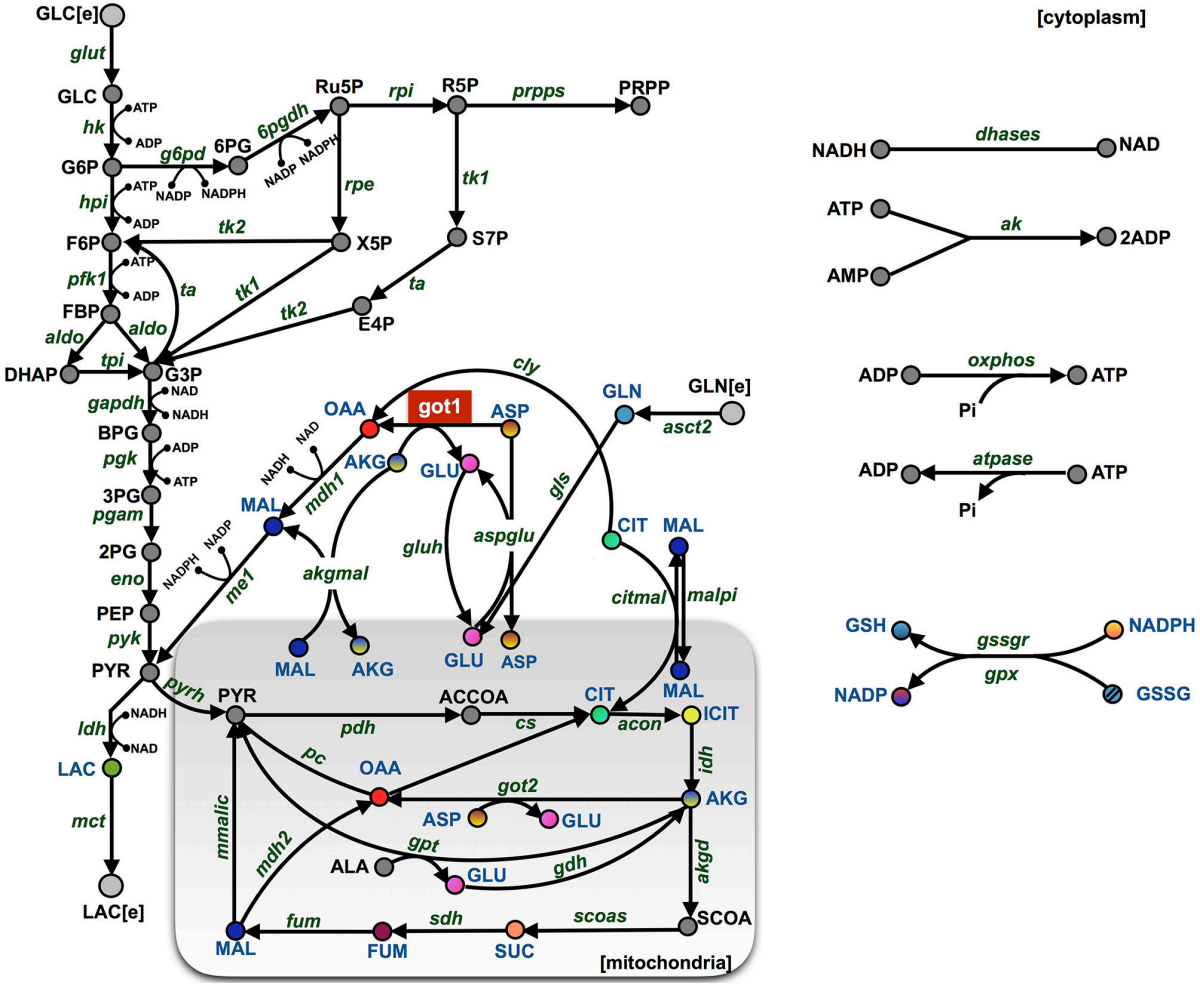
**Model schematic**. The metabolic network is comprised of 46 metabolites interacting through 53 enzymatic reactions. The major pathways involve glycolysis, glutaminolysis, the TCA cycle, the PPP, and shuttle reactions between mitochondrial (shaded rectangle) and cytoplasmic compartments. The abbreviations for the metabolites, cofactors, and enzymes are given in Supplementary File S3. The colored nodes represent the metabolites for which the fold-change has been measured experimentally during the knockdown of enzyme GOT1 (shown in red). The arrows represent the direction of the reaction fluxes in the baseline model at the initiation of the simulation.

We constructed a kinetic model of pancreatic cancer cell metabolism using previously published models of metabolism from various cell types (Mulukutla et al., 2010; Marín-Hernández et al., 2011; Mulukutla et al., 2012; Marín-Hernández et al., 2014; Shestov et al., 2014; Mulukutla et al., 2015). Our model is comprised of a total of 46 metabolites and 53 enzymatic reactions including glycolysis, glutaminolysis, the TCA cycle, the PPP, and malate-aspartate-ketoglutarate-glutamate shuttles between the cytosolic and mitochondrial compartments (Figure 1). Each step in the metabolic pathway is modeled according to known enzymatic reactions, which include reaction mechanisms ranging from simple Michaelis-Menten to complicated random bi-bi kinetics, expressed as different mathematical formulations. Rate laws for each reaction mechanism are incorporated into a system of 46 nonlinear ordinary differential equations (ODEs) that describe how the metabolite concentrations evolve over time. There is a single ODE for each metabolite, representing the rate of change of the species concentration, which depends on the rates at which the species is produced and consumed in the reaction network. We used an implicit differential equation solver in MATLAB (Guide, 1998) to numerically integrate the equations and solve for the metabolite concentrations. This is a deterministic model, which simulates the concentrations in a homogeneous ensemble of cells that experience, on average, similar intra- and extra-cellular environmental conditions. By integrating the ODEs, we calculate the average dynamics of the cell population.

We briefly summarize the model equations below, and the full set of ODEs is provided in the Supplementary Material (model files: “S1.m” and “S2.xml”). Abbreviations for the metabolites and reaction names are given in Supplementary File S3 and the values of the fixed parameters are listed in Supplementary File S4. The detailed rate equations for glycolysis and their corresponding kinetic constants are primarily based on the glycolysis model for HeLa cells (Marín-Hernández et al., 2011, 2014). This glycolysis reaction network was extended to include reactions from the TCA cycle and PPP using kinetic rate laws and parameters from Mulukutla and coworkers (Wu et al., 2007; Mulukutla et al., 2010, 2012, 2015). Reactions that involve ATP consumption and production in the cytoplasm were defined as in the model of Shestov et al. (2014), and the ATP and ADP concentrations in mitochondrial compartment were kept constant as in Mulukutla et al. In addition, glutamine transport parameters were obtained from Pingitore et al. (2013).

AKT is a strong promoter of KRAS-mediated pancreatic cancer tumorigenicity (Asano et al., 2004) due to its influence on the rates of metabolic reactions in glycolysis. It is known that PDAC cells have increased glucose uptake (Ying et al., 2012), which is mediated by upregulation of specific glycolytic enzymes, including the glucose transporter-1 (GLUT1), hexokinase (HK), and lactate dehydrogenase A (LDHA). Additionally, AKT promotes increased glucose uptake by activating GLUT1, HK, and the phosphofructokinase (PFK) enzyme (Rathmell et al., 2003; Elstrom et al., 2004). We have incorporated the effect of AKT in our metabolic model, simulating AKT-mediated enhanced glycolytic activity. Specifically, the activities of the GLUT1, HK, and PFK enzymes (represented by their individual *V*_*max*_ values) are modeled to have 20% basal activity, while 80% of their activity is due to activation by AKT (Mosca et al., 2012; Mulukutla et al., 2012).

In order to predict how the intracellular metabolic pathways influence cell growth, we incorporate cell number with the enzyme-catalyzed reactions. Specifically, the model is augmented to include a 47th ODE that describes the time evolution of the number of cancer cells, *C*_*N*_. Cell growth is implemented as a logistic equation (Enderling and Chaplain, 2014) that accounts for the maximal carrying capacity of the tumor, *K*_*CC*_ (Equation 1).

(1) $$\begin{array}{r} \frac{d\left(C_{N}\right)}{dt}=\left[\lambda \left(1-\frac{C_{N}}{K_{CC}}\right)C_{N}\right]-\alpha_{d}C_{N} \end{array}$$

The number of cancer cells is directly linked to the metabolism, where the growth rate depends on the intracellular concentrations of three primary metabolites known to influence cell proliferation: glucose, glutamine and ATP (Venkatasubramanian et al., 2008; Zhu et al., 2012). The dependence on these three metabolites is modeled assuming Monod-type functions (Higuera et al., 2009) (Equation 2).

(2) $$\begin{array}{r} \lambda =\alpha_{atp}\left(\frac{ATP}{k_{ap}+ATP}\right)+\alpha_{glc}\left(\frac{Glc_{in}}{k_{gc}+Glc_{in}}\right)+\alpha_{gln}\left(\frac{Gln_{in}}{k_{gn}+Gln_{in}}\right) \end{array}$$

The growth and death parameters α_*atp*_, α_*glc*_, α_*gln*_, and α_*d*_ are in the units of min^−1^. The concentration parameters *k*_*gc*_, *k*_*gn*_, and *k*_*ap*_ have units of mM.

### 2.2. Initial conditions

We simulated the model with multiple sets of starting metabolite concentrations to identify the appropriate range of initial conditions. There is limited information regarding the initial intracellular metabolite concentrations in pancreatic cancer cells. Therefore, we allowed the initial concentration for each metabolite to vary within a specified range. We specified the concentration ranges based on published measurements obtained from various cell lines, including human cervical cancer (Marín-Hernández et al., 2011, 2014), diseased and surrounding normal tissue samples from stomach and colon cancer patients (Hirayama et al., 2009), breast cancer cell extracts (Le Guennec et al., 2012), PDAC cancer patient samples (Fontana et al., 2016) and mouse myeloma and CHO cell lines (Mulukutla et al., 2012, 2015). Additional uncertainty for pancreatic cancer cells was considered by increasing and decreasing the upper and lower bounds, respectively, by 20%. Due to the lack of measurements that distinguish the metabolite levels in different cellular compartments, the initial concentrations of metabolites that were present in both mitochondrial and cytosolic compartments were assumed to be the same. The ranges of metabolite concentrations given in Table 1 account for variability in literature measurements as well an additional uncertainty for unknown intracellular concentration of pancreatic cancer cell lines in particular.

**Table 1:** **Bounds for initial conditions used in the model simulations**.

**Metabolite**	**Lower (mM)**	**Upper (mM)**	**Metabolite**	**Lower (mM)**	**Upper (mM)**
GLC	2.5 × 10^0^	1.4 × 10^1^	GSH	9.9 × 10^−2^	3.4 × 10^0^
ATP	1.4 × 10^−2^	1.4 × 10^1^	mPYR	1.2 × 10^−2^	1.4 × 10^1^
G6P	6.1 × 10^−3^	2.1 × 10^0^	mAcCoA	1.7 × 10^−4^	1.7 × 10^−1^
ADP	2.6 × 10^−3^	4.8 × 10^0^	mCIT	6.4 × 10^−3^	1.2 × 10^0^
F6P	3.4 × 10^−4^	8.4 × 10^−1^	mICIT	1.0 × 10^−2^	5.6 × 10^−2^
FBP	8.5 × 10^−3^	4.5 × 10^−1^	mAKG	5.8 × 10^−3^	2.3 × 10^−2^
DHAP	2.9 × 10^−3^	1.2 × 10^0^	mSCoA	1.6 × 10^−1^	3.0 × 10^0^
G3P	8.0 × 10^−4^	1.2 × 10^0^	mSUC	1.7 × 10^−1^	2.8 × 10^0^
NAD	1.8 × 10^−2^	2.2 × 10^0^	mFUM	1.7 × 10^−2^	2.2 × 10^−1^
13BPG	8.0 × 10^−4^	1.2 × 10^−1^	mMAL	9.6 × 10^−2^	2.4 × 10^0^
3PG	8.4 × 10^−3^	4.9 × 10^−1^	mOAA	9.6 × 10^−2^	2.4 × 10^0^
2PG	5.6 × 10^−3^	6.0 × 10^−2^	mASP	2.3 × 10^−1^	7.8 × 10^0^
PEP	1.8 × 10^−3^	3.8 × 10^−1^	mGLU	5.6 × 10^−3^	6.6 × 10^0^
PYR	1.2 × 10^−2^	1.4 × 10^1^	ASP	2.3 × 10^−1^	7.8 × 10^0^
LAC	8.0 × 10^−2^	7.3 × 10^1^	GLU	5.6 × 10^−3^	6.6 × 10^0^
AMP	3.6 × 10^−5^	3.4 × 10^0^	OAA	9.6 × 10^−2^	2.4 × 10^0^
6PG	3.2 × 10^−3^	1.1 × 10^−2^	MAL	9.6 × 10^−2^	2.4 × 10^0^
Ru5P	9.4 × 10^−3^	7.8 × 10^−2^	AKG	5.7 × 10^−3^	2.3 × 10^−2^
Xyl5P	8.0 × 10^−5^	1.9 × 10^−2^	CIT	6.4 × 10^−3^	1.2 × 10^0^
R5P	3.0 × 10^−3^	2.2 × 10^−2^	GLN	1.6 × 10^−1^	5.6 × 10^0^
E4P	8.0 × 10^−5^	2.7 × 10^−1^	NADH	8.0 × 10^−4^	1.0 × 10^−1^
S7P	6.5 × 10^−3^	8.1 × 10^−2^	NADPH	9.6 × 10^−4^	6.9 × 10^−2^
NADP	3.7 × 10^−3^	4.4 × 10^−1^	GSSG	1.0 × 10^−1^	1.1 × 10^0^

Latin Hypercube Sampling (McKay et al., 2000; Oguz et al., 2013) was applied to sample within the ranges selected for each metabolite. LHS separates the range of concentrations for the metabolites into multiple intervals and samples from each interval exactly once, thereby efficiently exploring the entire possible range of initial conditions for each metabolite. We selected to obtain 100 sets of initial conditions for each metabolite for parameter identifiability analysis (Section 3.1.1), and then randomly selected 50 of those sets to be used in parameter estimation (Section 3.1.3). This procedure adequately explores the possible ranges of initial conditions while balancing the computational resources required for global parameter optimization.

### 2.3. Parameter estimation

The baseline model, adapted from literature, has a total of 372 parameters, which includes 71 reaction velocities (the forward and reverse rates, *V*_*f*_ and *V*_*r*_, respectively). The reaction velocities reflect the amount of enzyme present and the corresponding enzyme activity. Conventionally, the reaction velocities are thought to distinguish the metabolism across different cell types. Therefore, of the many kinetic parameters included in the reaction rate equations, only the reaction velocities were fit to the training data, and the other rate constants were held at their literature values. We also fit the cell growth parameters shown in Equations (1) and (2). Below, we describe the experimental data used to train the model and the method used to perform the parameter estimation.

The model is fit to experimental measurements from Son et al. (2013), who measured the concentrations of 14 intracellular metabolites using targeted metabolomic analysis. Son and coworkers sought to understand the non-canonical glutamine metabolism in pancreatic cancer cells following the knockdown of GOT1, a major enzyme in glutamine metabolism. The metabolite concentrations were measured when the GOT1 enzyme was knocked down, relative to the no knockdown condition. Thus, they quantified the fold-change in the metabolite concentrations.

The experimental protocol used by Son and coworkers is illustrated in Figure S1. We simulated the same sequence of steps to predict the fold-change in the concentrations of the 14 metabolites. Since the relative enzyme expression level can be correlated with the regulation of enzyme activity levels, we simulate enzyme knockdown by multiplying the *V*_*f*_ by the factor (1 - α) (Nolan and Lee, 2012). We take the value of α to be 0.85, based on the average GOT1 expression level from two knockdown experiments reported in Son et al. (2013). The model is simulated for GOT1 knockdown to predict the fold-change in the concentration of the 14 metabolites relative to the no knockdown case. We sought to minimize the weighted sum of the squared error (WSSR) between the experimental data and the model predictions.

Additionally, Son and colleagues use *in vitro* cell culture to investigate how intracellular metabolism influences cell proliferation. They measure the number of cells with and without GOT1 knockdown and in the presence of varying extracellular nutrient concentrations. We also simulate their experimental protocols and compare the model predictions to their experimental measurements.

Particle swarm optimization (PSO) was used to identify the parameter values needed to enable the model predictions to best fit the data and minimize the WSSR. PSO (Iadevaia et al., 2010; Kennedy, 2010; Tashkova et al., 2011) is a biologically-inspired stochastic global optimization technique developed by Kennedy and Eberhart (1995). It is based on the concept of the social behavior observed in nature. In PSO, many *particles*, sets of parameters, are constantly updated from their random starting values to identify the parameter values that best fit the experimental data. Each particle has a position, representing the location in the multi-dimensional parameter space, and a velocity with which it moves toward a local minimum in the WSSR. The particles communicate with one another to update their position and velocity, ultimately moving toward the global minimum in the WSSR. We used PSO to estimate the reaction velocities for the baseline model. Each particle represents a vector of all reaction velocities to be optimized where the initial parameter values are taken from a well sampled space with the given bounds. To specify the bounds, the reaction velocities were allowed to vary 100-fold up and down from their starting values (taken from the literature, see Materials and Methods). Each run of the PSO algorithm executes 2, 500 iterations, a user-defined value to balance the computational expense of the parameter search. We performed the parameter estimation twice for each set of initial conditions (i.e., a total of 5, 000 iterations per initial condition) and, for each case, selected the set of parameters that generated the lowest error. This gave a total of 50 best-fit parameter sets, one set for each initial condition.

Estimating the reaction velocities for each initial condition was the first step of model fitting. In the second step of model fitting, we sought to estimate the growth parameters by minimizing the WSSR. Since there fewer parameters to fit compared to the first fitting step, we used nonlinear least squares optimization. We performed the fitting 100 times for each initial condition to approach the global minimum in the model error. Given limited prior knowledge of the range of base values for growth parameters (Higuera et al., 2009), we searched over a parameter space spanning seven orders of magnitude for each parameter. The model simulations to optimize for cell growth were conducted such that the same set of seven growth parameters could fit the experimental growth curve for both no knockdown and GOT1 knockdown conditions.

### 2.4. Data extraction

Experimental data for model training and validation was extracted from Son et al. (2013) using the MATLAB GRABIT program (Guide, 1998). Training data includes the fold-change in metabolite concentrations and cell number under GOT1 knockdown. Validation data includes the cell number under nutrient deprivation.

### 2.5. Parameter identifiability analysis

We used structural parameter identifiability analysis (Maly and Petzold, 1996; Ascher and Petzold, 1998; Shampine et al., 1999; Finley et al., 2011; Berthoumieux et al., 2013) to reduce the number of model parameters being fit to the training data. Parameter identifiability determines implicit dependencies among parameters. If two parameters are found to be correlated, we can specify a mathematical relationship between the parameters and only fit one in the parameter estimation procedure. Here, we only specify the relationship between correlated forward and reverse reaction velocities, where the reverse reaction velocity, *V*_*r*_, is expressed as a function of the forward reaction velocity, *V*_*f*_, with the equilibrium constant, *V*_*eq*_: *V*_*r*_ = *V*_*f*_/*V*_*eq*_. In these cases, only the forward reaction velocity is fit to the experimental data, thereby reducing the number of fitted parameters. The *V*_*eq*_ is calculated using the published works from which our model is derived (Wu et al., 2007; Mulukutla et al., 2010, 2012, 2015; Marín-Hernández et al., 2011, 2014).

### 2.6. Sensitivity analysis

We applied global sensitivity analysis (Saltelli et al., 2008) to determine which of the model parameters most significantly influence the predicted metabolite concentrations. Specifically, we used the extended Fourier Amplitude Sensitivity Test (eFAST) method (Marino et al., 2008), a variance-based approach, to understand the robustness of the model outputs (metabolite concentrations) given variance in the model inputs (the reaction velocities) (Zi, 2011). We allowed the model inputs to vary two orders of magnitude up and down from their literature values. The eFAST method calculates two indices that provide an estimate of the sensitivity of the model outputs with respect to the model parameters. The first order index, *S*_*i*_, quantifies the variance of the model output with respect to the variances of each individual input, and the total FAST index, *S*_*ti*_, quantifies the variance of the model output with respect to the variances of each input and covariances between combinations of inputs. The *S*_*i*_, then, is a measurement of local sensitivity of the model output to each individual input, whereas *S*_*ti*_ is a measure of the global sensitivity, accounting for the interactions or correlations among multiple inputs.

## 3. Results

We have constructed a kinetic model that predicts the dynamics of cellular metabolism in pancreatic cancer cells. The model is based on *a priori* knowledge of the molecular species involved and the reactions and interactions between the species. The complete model describing the metabolic network dynamics incorporates enzymatic reactions involved in glycolysis, glutaminolysis, the TCA cycle, and the PPP (Figure 1). We represent the cell using a cytoplasmic compartment and the mitochondria. Through glycolysis, glucose is metabolized to pyruvate, which enters the tricarboxylic acid cycle (in the mitochondria), or pyruvate can form lactate (in the cytoplasm), which is excreted from the cell. Glycolysis and pentose phosphate pathway take place in the cytoplasm and are linked through three metabolites: G6P, F6P and G3P. The TCA cycle in the mitochondrial compartment takes the influx of cytoplasmic pyruvate from glycolysis. Additionally, the following metabolites are exchanged between the cytoplasm and the mitochondria: malate, aspartate, citrate, glutamate and alpha-ketoglutarate. In total, the model includes 46 metabolites interacting through 53 enzymatic reactions where the evolution of the metabolites' concentrations are calculated by solving a set of nonlinear ODEs. The complete set of model reactions and the baseline parameter values from literature are included in the Supplementary Material.

### 3.1. Training of the complete kinetic model

We performed parameter estimation to fit the model to quantitative experimental data and estimate the reaction velocities (*V*_*f*_ and *V*_*r*_) that allow the model predictions to best match the available experimental data. As described in the Methods, the complete model is constructed using equations from multiple sources, each of which contains parameters that characterize the rates of the metabolic reactions. Therefore, we fit the model to data specific to pancreatic cancer in order to obtain a validated model that can be used to predict the dynamics of metabolism in pancreatic cancer cells.

#### 3.1.1. Parameter identifiability analysis

We first performed parameter identifiability (PI) to determine the pairs of correlated parameters. Specifically, we aimed to identify which of the total 71 forward and reverse reaction velocities are mathematically correlated. Completing this analysis allowed us to fit the forward rate, and calculate the reverse rate using the equilibrium constant. Initially 100 sets of initial conditions are chosen from Latin Hypercube Sampling. We sum the calculated correlation coefficients for each of the 100 initial conditions and subsequently normalized the estimated correlation coefficients. When the forward and reverse reaction velocities (*V*_*f*_ and *V*_*r*_, respectively) for a particular reaction are shown to be highly correlated for multiple sets of initial conditions, we fit the *V*_*f*_ and calculate *V*_*r*_ using the equilibrium constant, *V*_*eq*_. We performed the PI analysis once using the baseline model and all 71 reaction velocities, identifying 10 correlated pairs (“round 1”). We then performed the analysis again, after specifying the *V*_*r*_ values found to be correlated in round 1, which identified another two correlated pairs (“round 2"). Through this analysis, we reduced the number of reaction velocities to be fit from 71 to 59. The results of the parameter identifiability are shown in Figures S2–S4.

#### 3.1.2. Global sensitivity analysis

Next, we performed global sensitivity analysis to determine which of the reaction velocities most significantly influence the model outputs. Ideally, estimating the sensitivity of the predicted concentrations of the 14 metabolites to variance in the reaction velocities reduces the number of fitted parameters, where only the values of the most influential parameters are estimated. Therefore, we applied the eFAST method (see Section 2) to calculate the sensitivity of the fold-change in the metabolite concentrations given variance in the 59 reaction velocities included in the model, for each set of initial conditions. The cumulative result of the sensitivity analysis is shown in Figure S5, where we sum the sensitivity coefficients for the 50 sets of initial conditions. However, the results show that each of the parameters influence at least one of the predicted fold-changes for each set of initial conditions. Therefore, we moved forward with fitting all 59 parameters, so as not to omit any parameter that affects the predicted fold-changes.

#### 3.1.3. Parameter estimation

**Figure 2:**
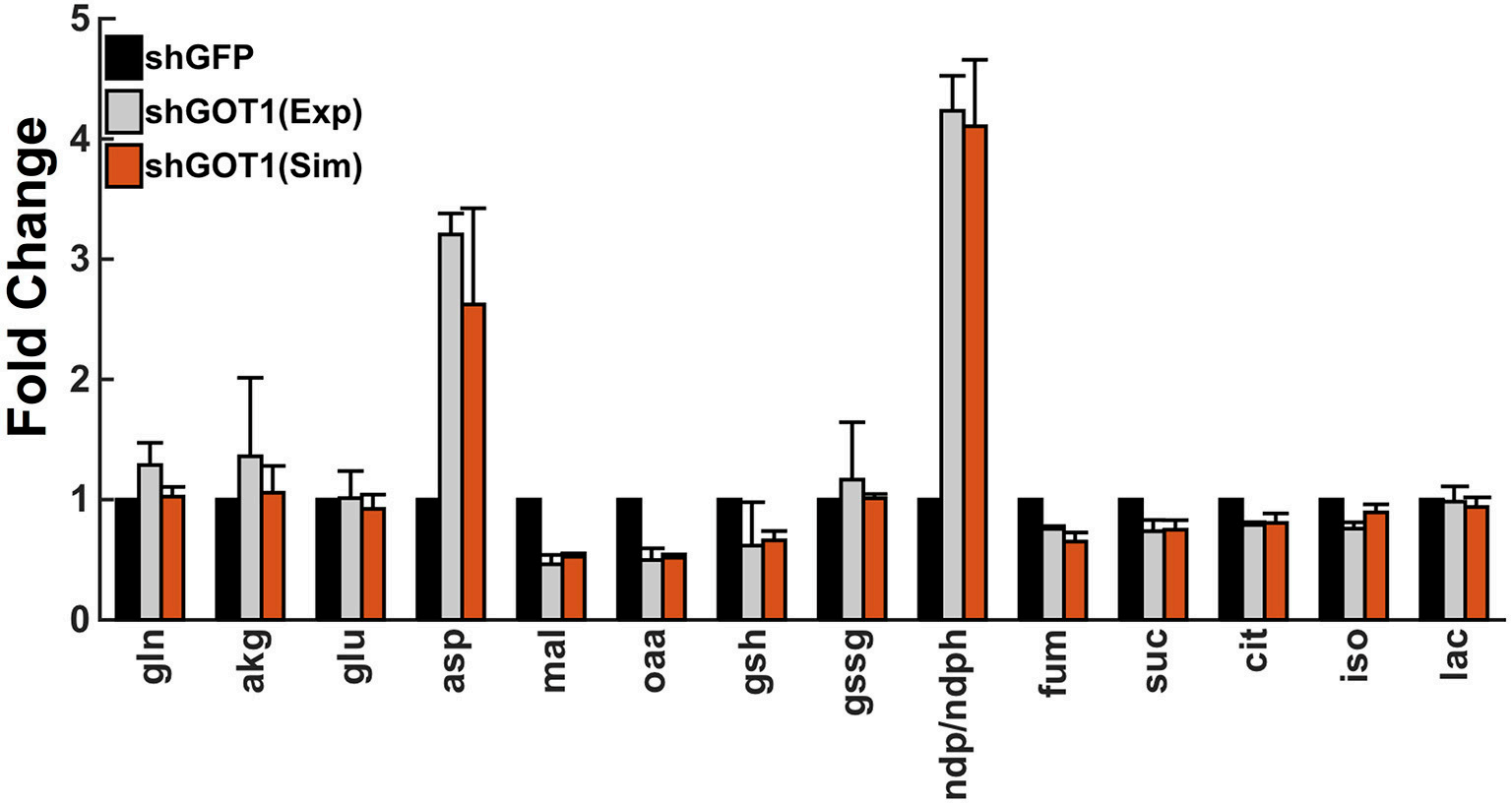
**Model fit to experimental data**. The model predictions for the fold-change in the metabolite concentrations (orange bars) match the experimental measurements from Son et al. (2013) (gray bars). Error bars for the predicted fold-change represent the standard deviation in the model predictions for the best fit from each of the 50 initial conditions. The simulated values of the metabolites derived from the mitochondria and cytoplasm are summed together to determine the total cellular metabolite pool, which was measured in the experiments.

Finally, we used particle swarm optimization (PSO) to find the optimal values for each reaction velocity that allow the fold-changes in the metabolite concentrations predicted by the model to accurately match the fold-changes measured experimentally. By performing the model training, the predicted fold-changes match very closely to the experimental data, as shown in Figure 2. As a result, we estimated the values of the reaction velocities for each set of initial conditions. The estimated parameter values are given in the Supplementary Material (“S5.xlsx”).

**Figure 3:**
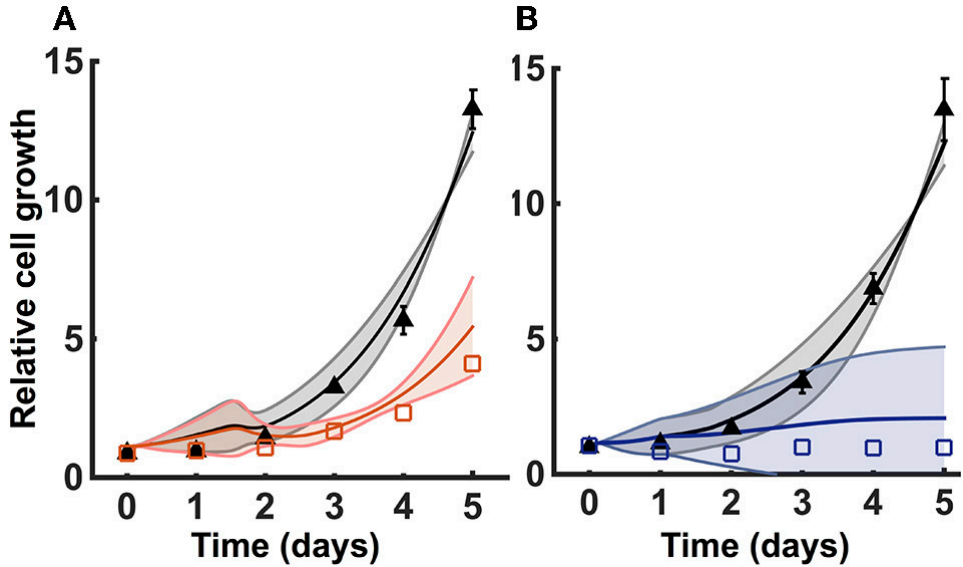
**Model training and validation using cell proliferation data**. The model is used to estimate the relative number of cells. **(A)** Simulated cell proliferation with complete media (35 mM glucose and 6 mM glutamine) for 5 days with no knockdown (black) and under GOT1 knockdown (red). Results are shown for the four initial conditions that match the training data. **(B)** Simulated cell proliferation when cells are grown in complete media for 5 days (black) or in complete media for 24 h, followed by glucose and glutamine deprivation for 4 days (blue). Results are shown for the final two initial conditions that match the validation data. In both **(A,B)**, triangles and squares represent the experimental data with error bars as available. The solid lines indicate the mean of predicted results for the given sets of initial conditions, and the shading shows the standard deviation.

We incorporated growth kinetics with the trained metabolic model to predict the number of cells over time. The cell growth is simulated in the presence of complete media (35 mM of glucose and 6 mM of glutamine) for a total time period of 5 days. The model is able to match the training data for the growth curves measured by Son et al. (2013) (Figure 3A). By training the model, we estimated the cell death rate and growth parameters that characterize how the concentrations of glucose, glutamine, and ATP contribute to the rate of cell proliferation (Equations 1 and 2). As a result, four initial conditions out of the total 50 starting initial conditions obtained from LHS were able to fit the data equally well (Figure 3A).

### 3.2. Model validation

We validated the model with available experimental measurements for cell proliferation under conditions of nutrient deprivation. The validation step confirms that the model is able to predict data not used in the model training. Two initial conditions with their corresponding fitted parameters (reaction velocities and growth parameters) could successfully validate the experimental growth curves measured under minimal glucose and glutamine concentrations (Figure 3B). These validated sets of initial conditions (Table 2) represent physiologically possible intracellular levels of metabolites present in pancreatic cancer cells. We therefore used only these sets of initial conditions and their corresponding fitted parameters in simulating various conditions and generating predictions that provide novel insight into pancreatic cancer metabolism.

**Table 2:** **Final sets of initial conditions that fit the training data and match the validation data well**.

**Metabolite**	**IC #1 (mM)**	**IC #2 (mM)**	**Metabolite**	**IC #1 (mM)**	**IC #2 (mM)**
GLC	1.4 × 10^1^	3.1 × 10^0^	GSH	2.1 × 10^0^	3.1 × 10^0^
ATP	7.7 × 10^0^	9.9 × 10^0^	mPYR	7.9 × 10^0^	6.9 × 10^0^
G6P	1.2 × 10^0^	1.8 × 10^0^	mAcCoA	1.2 × 10^−1^	1.0 × 10^−1^
ADP	1.9 × 10^0^	1.0 × 10^0^	mCIT	9.8 × 10^−2^	5.0 × 10^−1^
F6P	2.1 × 10^−1^	2.8 × 10^−2^	mICIT	1.7 × 10^−2^	2.49 × 10^−2^
FBP	1.0 × 10^−1^	3.8 × 10^−1^	mAKG	2.0 × 10^−2^	1.57 × 10^−2^
DHAP	8.8 × 10^−1^	7.7 × 10^−1^	mSCoA	7.2 × 10^−1^	1.5 × 10^0^
G3P	5.7 × 10^−1^	4.4 × 10^−2^	mSUC	2.5 × 10^0^	1.8 × 10^0^
NAD	3.1 × 10^−1^	8.1 × 10^−1^	mFUM	1.6 × 10^−1^	1.6 × 10^−1^
13BPG	6.7 × 10^−3^	1.4 × 10^−2^	mMAL	1.2 × 10^0^	2.2 × 10^0^
3PG	3.4 × 10^−1^	9.9 × 10^−2^	mOAA	1.9 × 10^0^	1.7 × 10^0^
2PG	4.9 × 10^−2^	4.5 × 10^−2^	mASP	4.3 × 10^0^	3.2 × 10^0^
PEP	5.4 × 10^−2^	2.1 × 10^−1^	mGLU	2.0 × 10^0^	3.0 × 10^−1^
PYR	8.1 × 10^0^	4.4 × 10^0^	ASP	7.0 × 10^0^	6.7 × 10^0^
LAC	1.9 × 10^1^	6.3 × 10^1^	GLU	2.8 × 10^0^	3.2 × 10^0^
AMP	2.5 × 10^−1^	1.3 × 10^0^	OAA	1.2 × 10^0^	1.2 × 10^0^
6PG	4.5 × 10^−3^	9.4 × 10^−3^	MAL	1.9 × 10^0^	2.1 × 10^0^
Ru5P	2.7 × 10^−2^	7.6 × 10^−2^	AKG	6.1 × 10^−3^	2.1 × 10^−2^
Xyl5P	1.3 × 10^−2^	1.3 × 10^−2^	CIT	5.1 × 10^−1^	1.7 × 10^−1^
R5P	1.4 × 10^−2^	6.0 × 10^−3^	GLN	4.3 × 10^0^	5.5 × 10^0^
E4P	1.8 × 10^−1^	5.2 × 10^−2^	NADH	6.1 × 10^−2^	9.7 × 10^−3^
S7P	5.7 × 10^−2^	7.1 × 10^−2^	NADPH	5.6 × 10^−3^	3.3 × 10^−2^
NADP	3.8 × 10^−1^	7.2 × 10^−2^	GSSG	3.2 × 10^−1^	9.1 × 10^−1^

The best-fit parameter sets estimated using these two initial conditions are remarkably consistent. A total of 69 and 71% of the reaction velocities and growth parameters, respectively, are within 100-fold of one another, as highlighted in Supplementary File S3. This consistency confirms the robustness of the identified parameter values and their physiological possibility within the intracellular environment of a pancreatic cancer cell, which is difficult to determine experimentally. However, given the large number of parameters that needed to be optimized, along with their interdependence due to upstream and downstream metabolite concentrations, some parameters showed high variability, as is common in systems biology models. Specifically, two growth parameters, α_*glc*_ and *k*_*gc*_, vary as widely as seven orders of magnitude between the two sets of best-fit parameter values estimated using the two validated initial conditions. These parameters characterize the contribution of glucose to the overall rate of cell proliferation. However, the ratio of α to *k* for glucose is very similar across the two sets of initial conditions, again pointing to the robustness of the estimated parameter values. The occurrence of high variability in the best-fit parameters is to be expected in highly nonlinear and complex kinetic models (Bellu et al., 2007). However, the strength of the model optimization lies in the fact that despite high variability in certain parameters, the model validation for both initial conditions is highly comparable, as evident from Figure S6.

### 3.3. Model robustness

**Figure 4:**
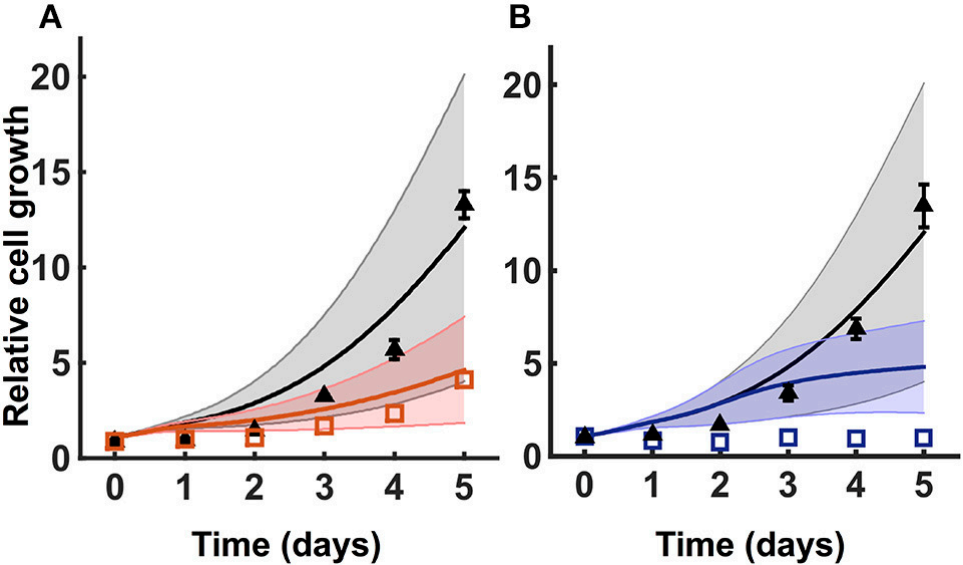
**Model robustness**. Model simulation using 1,000 random initial conditions selected from a Gaussian distribution, as described in Section 3.3. **(A)** Simulations under no knockdown (black) and GOT1 knockdown (red). **(B)** Simulations with complete media (black) and nutrient deprivation after 24 h (blue). In both **(A,B)**, triangles and squares represent the experimental data with error bars as available. The solid lines indicate the mean of predicted results for 1,000 sets of initial conditions, where the shading shows the standard deviation.

To test the robustness of the model predictions, we predicted how the number of cancer cells increase for varying metabolite initial conditions. We performed a Monte Carlo analysis, running the model with 1,000 different values of initial conditions randomly selected from a Gaussian distribution. The baseline initial condition for each metabolite is allowed to vary 50% up and down. Here, the mean is the baseline value for the initial condition, and the standard deviation is 1/6 of the mean. This ensures that all of the values selected from the Gaussian distribution are within three standard deviations of the mean. The predicted results for one of the validated sets of initial conditions are shown in Figure 4. The simulations indicate that cell proliferation is fairly sensitive to the initial metabolite concentrations. Therefore, our careful procedure of identifying an appropriate set of initial conditions is important in generating valid model predictions.

### 3.4. Predicted effects of nutrient availability

**Figure 5:**
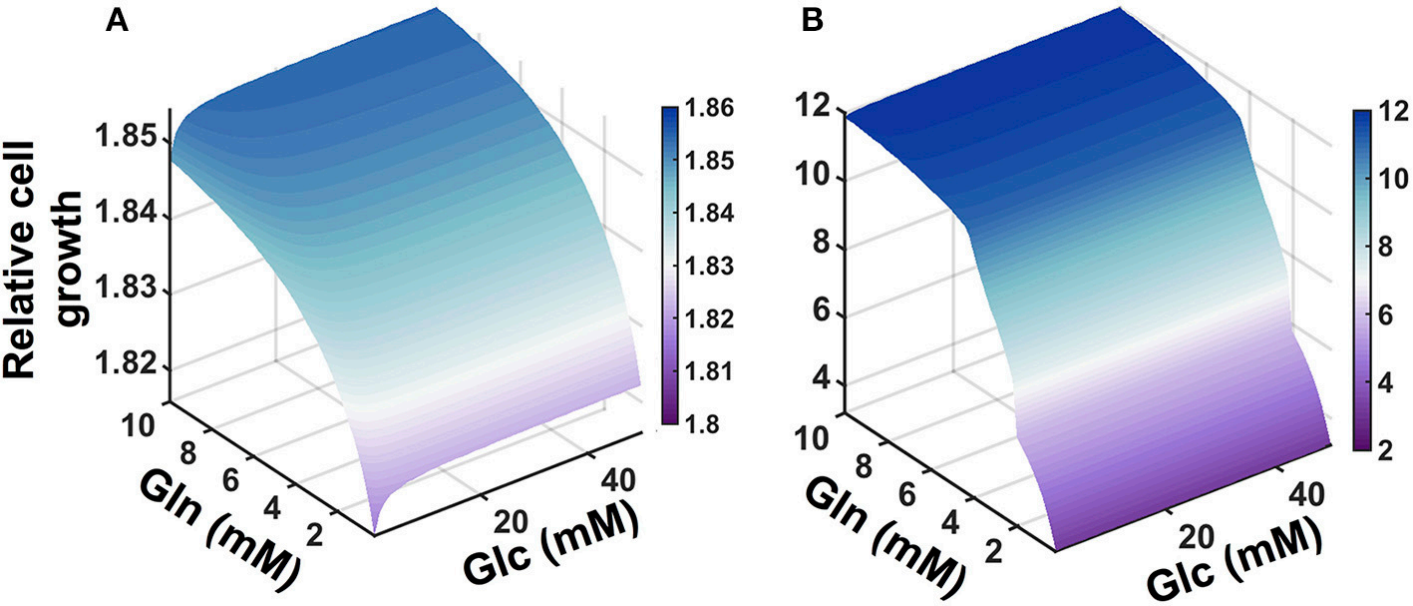
**Predicted effects of varying nutrient availability**. Predicted relative number of pancreatic cancer cells with varying extracellular concentrations of glucose and glutamine at: **(A)** 1 day and **(B)** 5 days.

We applied the model to investigate the effects of the availability of glucose and glutamine in the extracellular environment. The cell proliferation rate is explicitly dependent on the concentrations of glucose and glutamine (Ramanathan et al., 2005; Yun et al., 2009), as well as the ability to convert the nutrient sources into ATP. Therefore, we explored how the cell count varied given changes in the extracellular levels of glucose and glutamine. We simulated the model under varying conditions of both glucose and glutamine (Figure 5). The model predicts that nutrient availability influences cell proliferation in a nonlinear manner. Additionally, the number of pancreatic cells is predicted to be more dependent on glutamine availability, as compared to glucose, particularly given longer times for cell growth. This result, which holds true for both validated sets of initial conditions, is consistent with experimental observations (Gaglio et al., 2011).

### 3.5. Predicted effects metabolic fluxes

**Figure 6:**
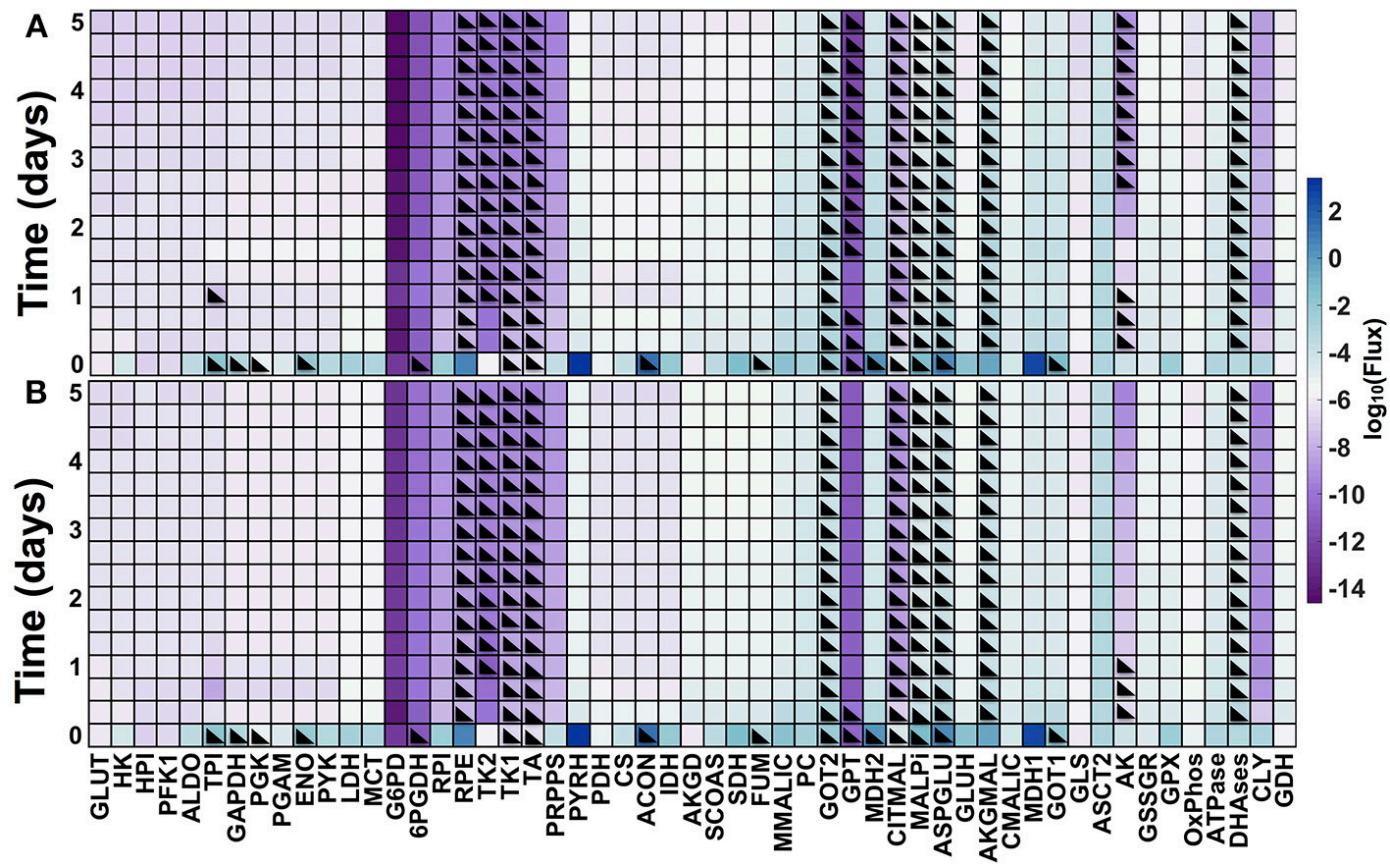
**Predicted metabolic fluxes**. Dynamic fluxes predicted by the model for: **(A)** no knockdown and **(B)** GOT1 knockdown. The color bar indicates the magnitude of the flux on a log scale. Black triangles denote the time points at which the flux is in the opposite direction (i.e., negative flux), compared to the baseline model shown in Figure 1.

The model predicts the dynamic reaction fluxes under varying conditions, providing insight into the metabolic phenotype of the pancreatic cancer cells. The flux through the enzyme-catalyzed reactions indicates the functional impact of each connection in the metabolic network (Sauer, 2006). Therefore, we applied the model to predict the dynamic reaction fluxes through the metabolic reactions both in the baseline model with no GOT1 knockdown (Figure 6A) and under GOT1 knockdown (Figure 6B). The differences in the reaction fluxes between these two conditions provide mechanistic insight into how altering a single enzyme-catalyzed reaction has a systemic effect on the metabolic network. The model predicts that GOT1 knockdown influences the magnitude and direction of the adenylate kinase (AK) reaction. The AK enzyme catalyzes the production of ADP from ATP and AMP, and in the baseline model, this reaction mostly proceeds in the reverse direction (i.e., there is a net production of ATP). With GOT1 knockdown, the flux through the AK reaction switches after 24 h of cell growth. In this case, less ATP is available to be consumed for proliferation, hence lower cell growth is observed. Additionally, GOT1 knockdown causes the glutamate-pyruvate transaminase (GPT) reaction to proceed in the opposite direction, as compared to the no knockdown case. This means that with GOT1 knockdown, the GPT reaction works to produce glutamate rather than consume it, compensating for the lower glutamate production that occurs when the GOT1 enzyme is not fully active.

### 3.6. Predicted response to metabolic perturbations

**Figure 7:**
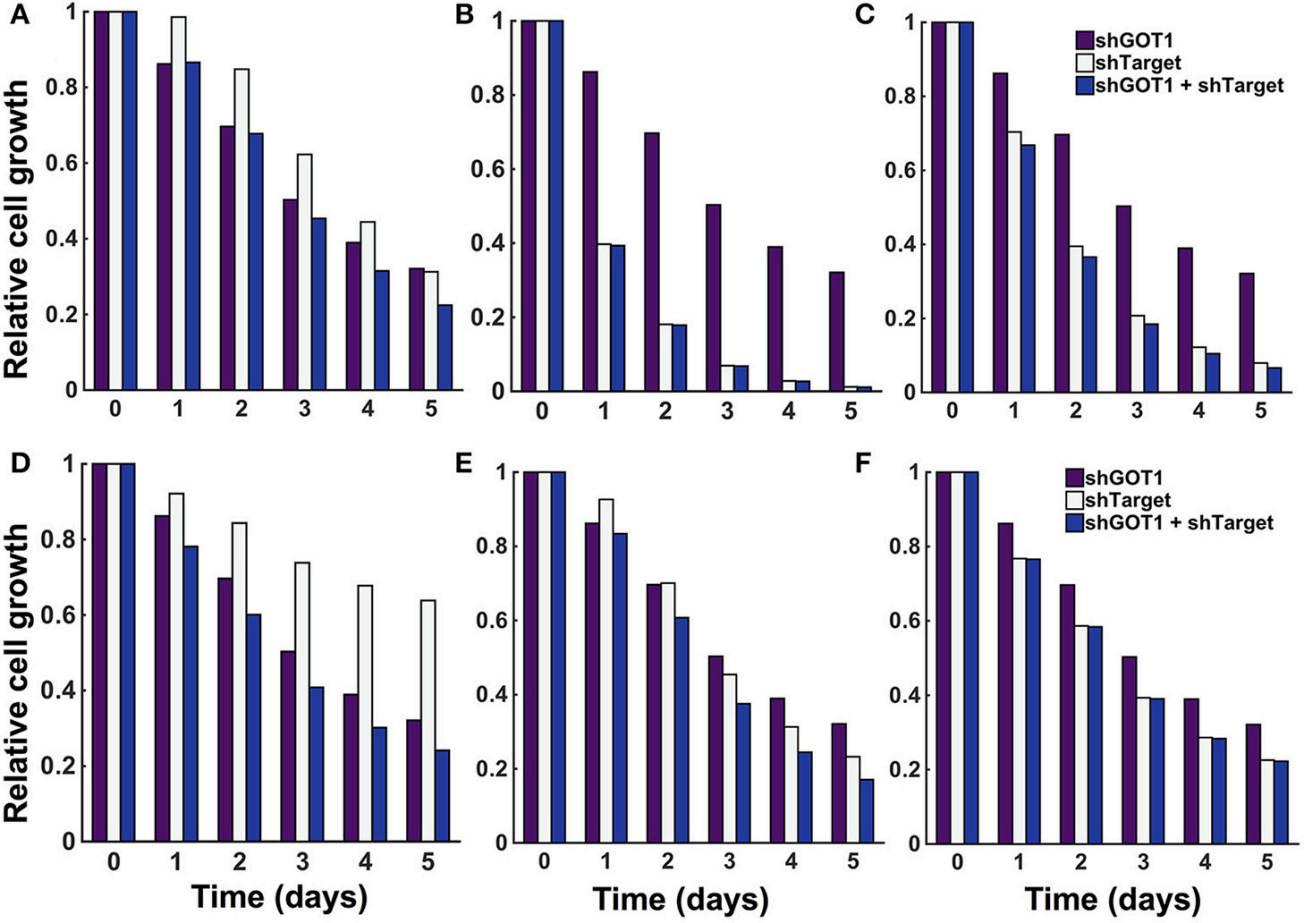
**Predicted response to metabolic perturbations**. The model predicts the relative number of pancreatic cancer cells when the activity of the target enzyme was reduced by 85% alone or in combination with 85% GOT1 knockdown. The targets investigated were **(A)** GLUT1, **(B)** OXPHOS, **(C)** GLS, **(D)** MALPi, **(E)** GAPDH, and **(F)** GOT2.

The model predicts the systems-level response to various metabolic perturbations. With the ability to predict the number of pancreatic cancer cells over time and the dynamic reaction fluxes, the model can help identify the enzyme-catalyzed reactions that are effective therapeutic targets to inhibit tumor metabolism and impede cell growth. Therefore, we applied the model to predict the effects of inhibiting various enzymes in the metabolic network. We implemented enzyme knockdowns by decreasing the forward reaction velocity (*V*_*f*_) by 85%, either alone or in combination with GOT1 knockdown. We first targeted enzymes that directly influence the three metabolites involved in the cell proliferation rate (glucose, glutamine, and ATP). These enzymes include GLUT1, which catalyzes glucose uptake by the cell, GLS, the enzyme that converts glutamine to glutamate, and OXPHOS, the reaction simulating oxidative phosphorylation. The model predicts that inhibiting these enzymes influences cell growth to varying degrees. GLUT1 knockdown alone is not as effective in reducing cell growth as GOT1 knockdown (Figure 7A). Moreover, knockdown of both GLUT1 and GOT1 is as effective in reducing cell growth as GOT1 knockdown alone. Thus, the model indicates that GLUT1 is not an optimal target, as compared to GOT1. In comparison, OXPHOS knockdown leads to lower cell proliferation compared to GOT1 knockdown (Figure 7B). Also, under GLS knockdown, cell growth is significantly reduced (Figure 7C), alone or in combination with GOT1 knockdown.

The model predicts novel strategies to reduce pancreatic cancer cell metabolism that lead to reduced cell proliferation. After targeting enzymes that directly influence the metabolites whose concentrations influence the cell proliferation rate, we examined the effects of altering other enzymes in the metabolic network, individually and in combination. We conducted a local sensitivity analysis by varying the reaction velocities and predicting the effects on the relative cell number. We systematically reduced each of the 59 fitted reaction velocities in the trained model from 5% knockdown up to complete knockout (Burgard et al., 2003; Meister et al., 2013). In this way, the model is used to specifically pinpoint which enzyme-catalyzed reactions contribute most to cell growth inhibition. Reducing the reaction velocity in the GOT1 reaction showed an expected direct correlation of decrease in cell growth with increasing effect of knockdown (Figure S7). However, it is more interesting to apply the model to identify combination therapies, i.e., systematic combinations of knockdown of essential enzymatic reactions. Therefore, we identified how knockdown (reducing the reaction velocity by 85%) for a target enzyme influences the predicted cell growth, alone and in combination with GOT1 knockdown. The model predicts three relevant classes of behaviors that lead to a reduction in cell proliferation, as described below. We show the relative cell count for a representative example from each case in Figure 7D, MALPi; Figure 7E, GAPDH; and Figure 7F, GOT2.

1. *Knockdown of the target enzyme alone is not as effective as GOT1, but its knockdown synergizes with GOT1 knockdown to further decrease cell count*. We identified the malate-phosphate shuttle (MALPi) as a representative example. MALPi is responsible for phosphate shuttle across the cytoplasmic and mitochondrial compartment and hence for the conversion of ATP and ADP. MALPi, in conjunction with the citrate malate shuttle (CITMAL), generates citrate required for lipid synthesis. Therefore, targeting MALPi exhibits is expected to reduce tumor growth, which the model predicts (Figure 7D).
2. *Knockdown of the target enzyme reduces cell proliferation as much as GOT1 knockdown alone, and is even more effective when combined with GOT1 knockdown*. This behavior is illustrated in the case of targeting GAPDH, the enzyme responsible for converting G3P to BPG, accompanied by the reduction of NAD to NADH. Interestingly, over-expression of GAPDH has been observed in many types of cancers (Norris et al., 2008; Ganapathy-Kanniappan et al., 2012; Krasnov et al., 2013). Inhibiting GAPDH would decrease the production of downstream metabolites, hence reducing the formation of lipids and amino acids, which are required for cell proliferation (Pereira et al., 2009). As expected, the model predicts reduced cell proliferation upon inhibiting the GAPDH enzyme (Figure 7E).
3. *Knockdown of the target enzyme alone is very effective in reducing cell proliferation, and combining it with GOT1 knockdown does not have any additional effect*. A representative example of this behavior is shown by targeting glutamate oxaloacetate transaminase 2 (GOT2). This enzyme promotes synthesis of OAA by AKG via glutamate. The expression level and activity of the GOT2 enzyme has been found to be highly elevated in pancreatic and breast cancer cells (Chakrabarti et al., 2015; Korangath et al., 2015; Yang et al., 2016). The model predicts that targeting GOT2 activity is a potential lethal approach to target glutamine metabolism to inhibit tumor growth (Figure 7F).

## 4. Discussion

### 4.1. Robust and predictive computational model

We present a predictive model that enables quantification of the kinetics of the intracellular metabolism of pancreatic cancer cells. The model provides an understanding of how the cells depend on the extracellular conditions (Vander Heiden et al., 2009) and the resulting dynamic reaction fluxes. The ultimate goal is to use the model to tackle this aggressive disease by identifying novel strategies to alter the reprogrammed metabolism within cancer cells (Hanahan and Weinberg, 2011).

The model is predictive of pancreatic cancer cell metabolism in particular, as we carefully calibrated the model to pancreatic cancer-specific data from the 8988T cell line. The calibrated model predicts the metabolite concentrations, reaction fluxes, and number of pancreatic cells over time. As a result of model calibration and validation to data not used in training, we identify feasible sets of initial conditions and kinetic parameters that together provide a model that is specific to pancreatic cancer. We apply the validated model to predict the effects of perturbing specific metabolic reactions, alone and in combination. Interestingly, the model simulations show that targeting the PPP, TCA cycle, or mitochondrial-cytoplasmic shuttle reactions presents an equally important and synergistic role with targets to regulate tumor metabolism.

Computational modeling offers a powerful tool to incorporate the complexity and robustness of the interconnected metabolic pathways and predict how individual and subsets of metabolic reactions give rise to the systemic behavior of the cells. Through parameter identification, sensitivity analyses, and parameter estimation, we obtained a predictive computational model that matches experimental data and can be used to predict metabolic phenotypes of pancreatic cancer. We utilized a quantitative approach to predict how altering nutrient availability and enzyme activity inhibits cancer cell metabolism, and ultimately, cancer cell proliferation. In this way, the model is a valuable framework that generates hypotheses regarding novel therapeutic strategies. The model provides quantitative insight into how the dynamics of metabolism are affected by strategic knockdown of enzyme activity. The strategies that we implemented computationally can be tested experimentally using shRNA to selectively reduce the activity of the targeted enzyme(s). Thus, when combined with experimental studies, the model can prove useful in designing and understanding pre-clinical trials.

Our approach of fitting the model with different sets of initial conditions to generate multiple parameter sets is akin to ensemble modeling for metabolic systems (Tran et al., 2008; Srinivasan et al., 2015; Saa and Nielsen, 2016). The ensemble modeling approach, which has been applied to build dynamic genome-scale models, generates multiple parameter sets (an ensemble of models) that produce the same steady state conditions. Given additional data, such as the distributions of the reaction fluxes under certain perturbations, the number of feasible models can be reduced. The ensemble of models is produced by sampling the parameter space for the kinetic rates, given certain constraints (i.e., thermodynamics or growth requirements). Analogously, we have sampled the space of possible initial metabolite concentrations and trained the model for each set of initial conditions to generate a set of possible kinetic parameters. We then use the cell proliferation data to further identify the sets of appropriate parameters and initial metabolite concentrations. This procedure resulted in two possible models, which are then evaluated to determine their robustness, and finally applied to generate novel predictions.

### 4.2. Comparison to other studies

The metabolic model constructed in this work is a significant expansion beyond existing kinetic models of cancer metabolism. Previously published kinetic models in the context of cancer have mostly focused on the glycolytic pathway. Such models have successfully identified enzymes that are associated with tumor growth and malignancy and are important targets in inhibiting metabolism, including GLUT, HK, PFK-1, and GAPDH (Marín-Hernández et al., 2011, 2014; Shestov et al., 2014). However, the enzymes involved in the TCA cycle and glutaminolysis also significantly contribute to cancer cell proliferation, particularly in case of pancreatic cancer. Our paper is the first to combine these pathways, along with cell growth, in a model for pancreatic cancer, thereby advancing the field of dynamic metabolic modeling of cancer. The impact of enzymes that catalyze glutaminolysis and TCA cycle reactions was proven experimentally by Son et al. (2013) and our simulations also confirm their importance.

We can compare the model predictions to experimental studies published in the literature. Over-expression of GLUT has been identified in almost all types of cancer and hence is a key signature of malignancy (Ganapathy-Kanniappan and Geschwind, 2013). Targeting GLUT has been shown to inhibit glucose transport and reduce cell growth(Liu et al., 2012; Granchi et al., 2014). However, due to the ubiquitous expression of GLUT in all cell types, blockage of GLUT remains a critical challenge. Using the model, we could successfully confirm the presence of alternative targets described in the literature, as well as identify novel targets. The model predicts the effects of targeting other pathways by which tumor cells metabolize nutrients and produce building blocks needed for cell proliferation. For example, the model predicts that targeting oxidative phosphorylation (via the OXPHOS enzyme) can significantly reduce cell growth, in combination with inhibition of the GOT1 enzyme. Indeed, the literature has shown that targeting this pathway by which the cell generates ATP in the mitochondria (Caro et al., 2012; Haq et al., 2013; Vazquez et al., 2013; Viale et al., 2014; Weinberg and Chandel, 2015), synergistically with optimal inhibition of glycolysis and glutaminolysis may increase effectiveness of cancer therapeutics (Lu et al., 2015; Yadav et al., 2015). Another example is inhibition of glutaminase (GLS), the enzyme responsible for converting glutamine to glutamate. The glutamate produced in this reaction subsequently enters in the TCA cycle to ultimately generate metabolites such as OAA, AKG, acetyl-CoA, and citrate for lipid production and nitrogen for DNA synthesis (Chen and Cui, 2015). The GLS enzyme is reported to have a positive correlation with cancerous tumor growth from normal cells due to enhanced glutaminolysis (Lora et al., 2004; Xiang et al., 2015), making it is a potential target for effective cancer therapeutic. The model predicts a synergistic effect when GLS is inhibited in combination with GOT1. Interestingly, inhibitors of GLS are being explored: BPTES (DeLaBarre et al., 2011; Hartwick and Curthoys, 2012) and CB839 (Gross et al., 2014) have been shown to induce apoptosis in cancer cells. These predicted effects of targeting OXPHOS and GLS, along with those described in Section 3.6 and illustrated in Figure 7 demonstrate the utility of the model and confirm its validity. Excitingly, this comparison of the model results and known experimental studies lends great confidence to the model's predictions.

### 4.3. Model limitations

Our model accurately reproduces, both quantitatively and qualitatively, experimental data used for training and validation. However, there are certain limitations that can be addressed as additional quantitative data become available for model fitting. Currently, the model only considers cancer cells; however, it is important to consider additional cell types within the tumor. We can extend the model to predict the effects of interactions between multiple cell types and to understand the dynamics of exchange of nutrients between the cells. Expanding the model in this way could enable a better understanding of the symbiosis between cells (Mendoza-Juez et al., 2012) and how the tumor microenvironment can alter the cells' metabolic dependencies and induce apoptosis (Phipps et al., 2015). Another limitation is that the model does not include intracellular recycling pathways or scavenging mechanisms such as autophagy (organelle degradation by autophagosomes) or macropinocytosis (engulfing the nutrients followed by lysosomal degradation). Additionally, the model assumes that the concentrations of glucose, glutamine, and ATP directly correlate to the cellular resources required for biomass production and cell proliferation. Therefore, we do not include the steps toward amino acid synthesis or nucleotide synthesis through the non-oxidative arm of the PPP or the hexosamine biosynthesis pathway. These are processes that enable cancer cells to promote biomass synthesis and could be added as future extensions to the existing model. Finally, given additional data, the model can be adapted to predict the metabolism in a range of cancer cell types beyond pancreatic cancer.

## 5. Conclusion

The metabolic model presented here is a novel computational tool for investigating the metabolism of pancreatic cancer cells. The model includes enzyme-catalyzed reactions in central metabolic pathways and is trained and validated using quantitative experimental measurements, specific to pancreatic cancer lines. As a result, we have constructed the first kinetic model of pancreatic cancer metabolism. The model predicts the effects of both intracellular and extracellular perturbations, providing the metabolic fluxes and the number of cancer cells over time. With a successful identification of appropriate initial conditions and parameter values for pancreatic cancer, the model serves as a good starting point to predict the dynamic metabolism in other pancreatic cancer cell lines as well as a template for studying cell growth in other cell types. Additionally, using model simulations, we can design novel *in silico* combinatorial therapies toward impeding cancer cell proliferation. Thus, the model can be used to complement *in vitro* and *in vivo* pre-clinical studies.

## Author contributions

SF designed the research. MR constructed the model and performed the simulations and analyses. All authors contributed to writing the manuscript and approved of its final version.

## Funding

This work is supported by The Rose Hills Foundation and the USC Provost's Office (research grant to SF).

### Conflict of interest statement

The authors declare that the research was conducted in the absence of any commercial or financial relationships that could be construed as a potential conflict of interest.
