# Supplementary material for: Computational Model Predicts the Effects of Targeting Cellular Metabolism in Pancreatic Cancer
Source: Front Physiol. 2017 Apr 12;8:217. doi: 10.3389/fphys.2017.00217 (PMC5388762; doi:10.3389/fphys.2017.00217)
Supplement: Supplementary file 1 [file Presentation1.PDF]

frontiers

Supplementary Material: Computational model predicts the effects of targeting cellular metabolism in pancreatic cancer

Mahua Roy [1] **and Stacey D. Finley** [1,2*]
*Correspondence:
Dr. Stacey D. Finley
sfinley@usc.edu

1 SUPPLEMENTARY DATA

Supplementary File S1. Rate equations for the reactions included in the kinetic metabolic model as MATLAB file. ("S1.m")
The model includes 46 metabolites, plus an additional species "cellnumber", which corresponds to the number of cells. There are 53 enzymatic reactions, and an additional reaction corresponding to logistic cell growth. Thus, in total, the model includes 47 species and 54 reactions. The model predicts the metabolite concentrations in units of mM (mmol/L).

Supplementary File S2. Rate equations for the reactions included in the kinetic metabolic model as SBML file. ("S2.xml")
The model includes 46 metabolites, plus an additional species "cellnumber", which corresponds to the number of cells. There are 53 enzymatic reactions, and an additional reaction corresponding to logistic cell growth. As described below, we include 12 additional reactions that for implementing transport reactions. Therefore, in total, the SBML implementation of the model includes 47 species and 66 reactionsThe model predicts the metabolite concentrations in units of mM (mmol/L).

Notes on model implementation:

1. *Initial conditions.* The initial metabolite concentrations correspond to IC#1 from Table 2. The initial number of cells is 2000.
2. *Transport reactions.* There are 12 additional reactions included in the SBML model due to the way in which we implemented transport reactions between the cytosol and mitochondria. Specifically, we implement the reversible transport for a species as two separate irreversible reactions. For example, the Pyruvate Hydrogen Shuttle reaction (PYRH) is:
   $cPYR \longleftrightarrow mPYR$

   We implement this reaction as:
   $cPYR \longrightarrow$ reaction rate = PYRH_reaction
   $\longrightarrow mPYR$ reaction rate = PYRH_reaction$*Vc/Vm$

   where PYRH_reaction is the reaction rate as defined in the model and Vc and Vm are the volumes of the cytosol and mitochondria, respectively. This allows us to explicitly account for the difference

in volumes between the two cellular spaces, rather than relying on the modeling software to do this. There are three reactions that involve one species being transported: PYRH, MALPi, and GLUH, giving an additional 3 reactions (one for each reaction). There are three reactions that involve two species being transported: ASPGLU, CITMAL, and AKGMAL, giving 9 additional reactions (three for each reaction). In total, this adds up to the additional 12 reactions. Given this implementation, the transport reactions are now seemingly independent, as there is no connection between the cytosolic and mitochondrial forms of a species that is transported between the two cellular spaces. Therefore, visualization of the model network will yield three disconnected networks corresponding to species in the extracellular space, cytosol, and mitochondria. The notation for the species' names is given in Supplementary File S3.

Supplementary File S3. Abbreviations for the metabolites and reactions included in the kinetic metabolic model. It includes KEGG ID's of each metabolite. The respective compartments are indicated as prefix - m for mitochondria ; c for cytosolic. All enzymatic reactions as well as the references for the reaction mechanisms are also provided, both in a general format as well as the way it is implemented in the model. ("S3.xlsx")

Supplementary File S4. Fixed rate constants for the kinetic metabolic model. The citation for each parameter value is also provided. ("S4.xlsx")

Supplementary File S5. Fitted reaction velocities for the kinetic metabolic model. ("S5.xlsx")

2 SUPPLEMENTARY FIGURES

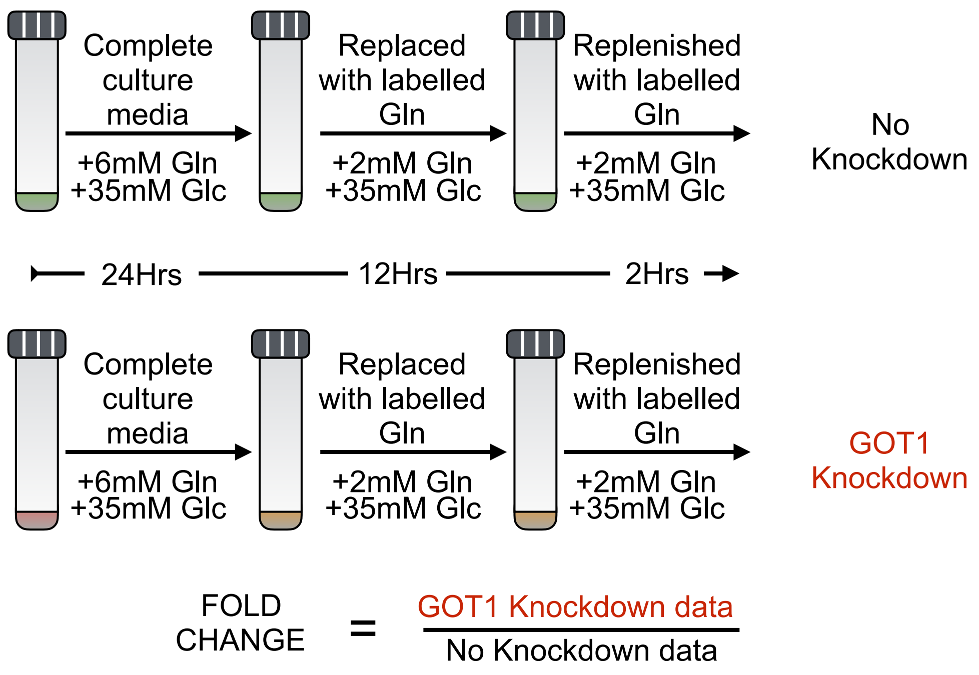

Figure S1. Schematic of the conditions simulated in the model. The model simulations mimic the experimental protocol followed by Son *et al.* The fold-changes in the concentrations of 14 metabolites predicted by the model are compared to the experimental data collected by Son *et al.* Labelled glutamine used in experiment is equivalent to unlabelled glutamine addition in simulations.

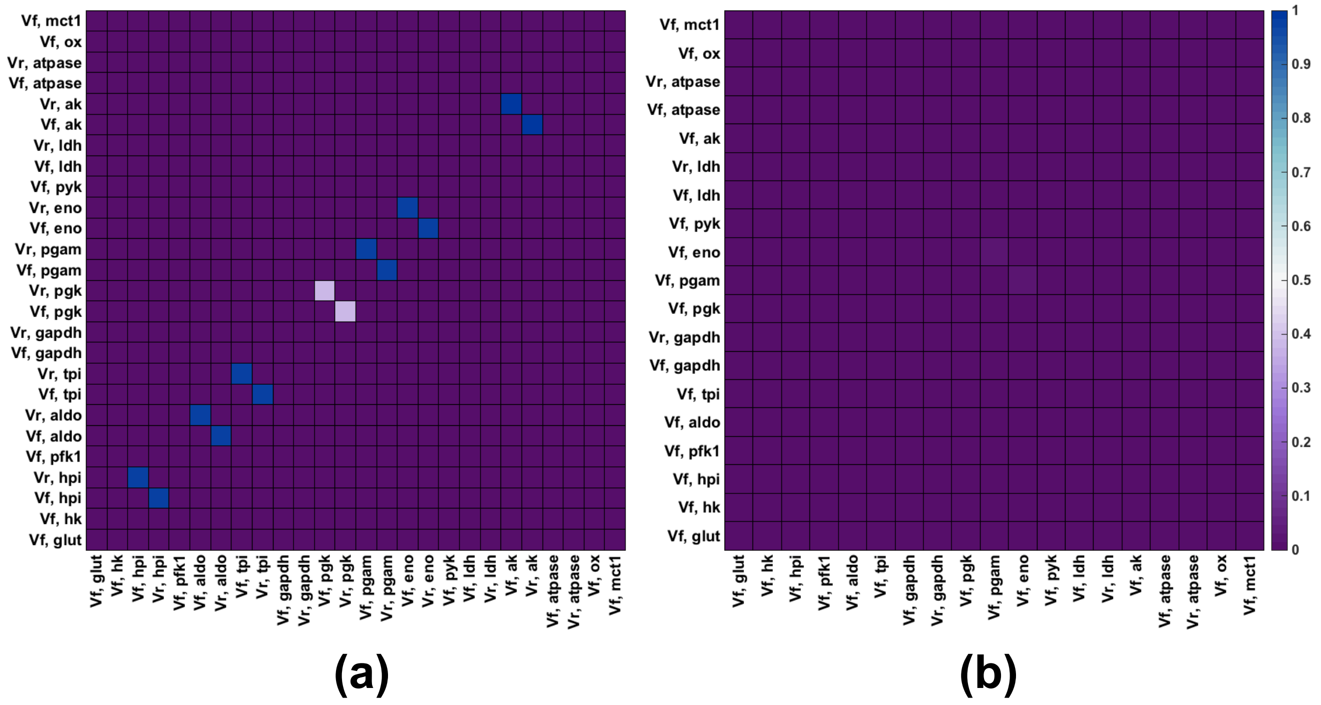

Figure S2. Results from the parameter identifiability analysis for reaction velocities involved in glycolysis Estimated correlation coefficients for **(a)** Round 1 and **(b)** Round 2. This analysis identified seven sets of correlated parameters (all identified in Round 1), enabling us to reduce the number of fitted reaction velocities from 26 to 19. The forward and reverse rates found to be correlated were for the following reactions: HPI, ALDO, TPI, PGK, PGAM, ENO, and AK.

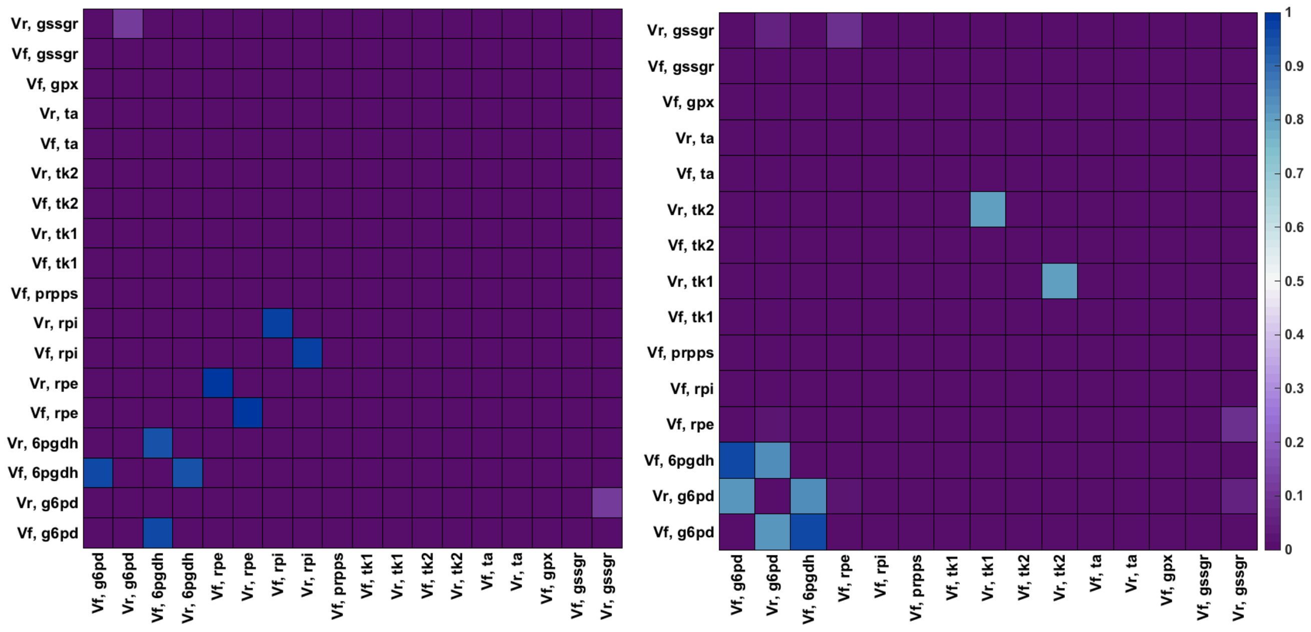

(a) **(b)**

Figure S3. Results from the parameter identifiability analysis for reaction velocities involved in the PPP Estimated correlation coefficients for **(a)** Round 1 and **(b)** Round 2. This analysis identified a total of four sets of correlated parameters, enabling us to reduce the number of fitted reaction velocities from 15 to 12. The forward and reverse rates found to be correlated were for the following reactions: from Round 1 - 6PDGH, RPE and RPI; from Round 2 - G6PD.

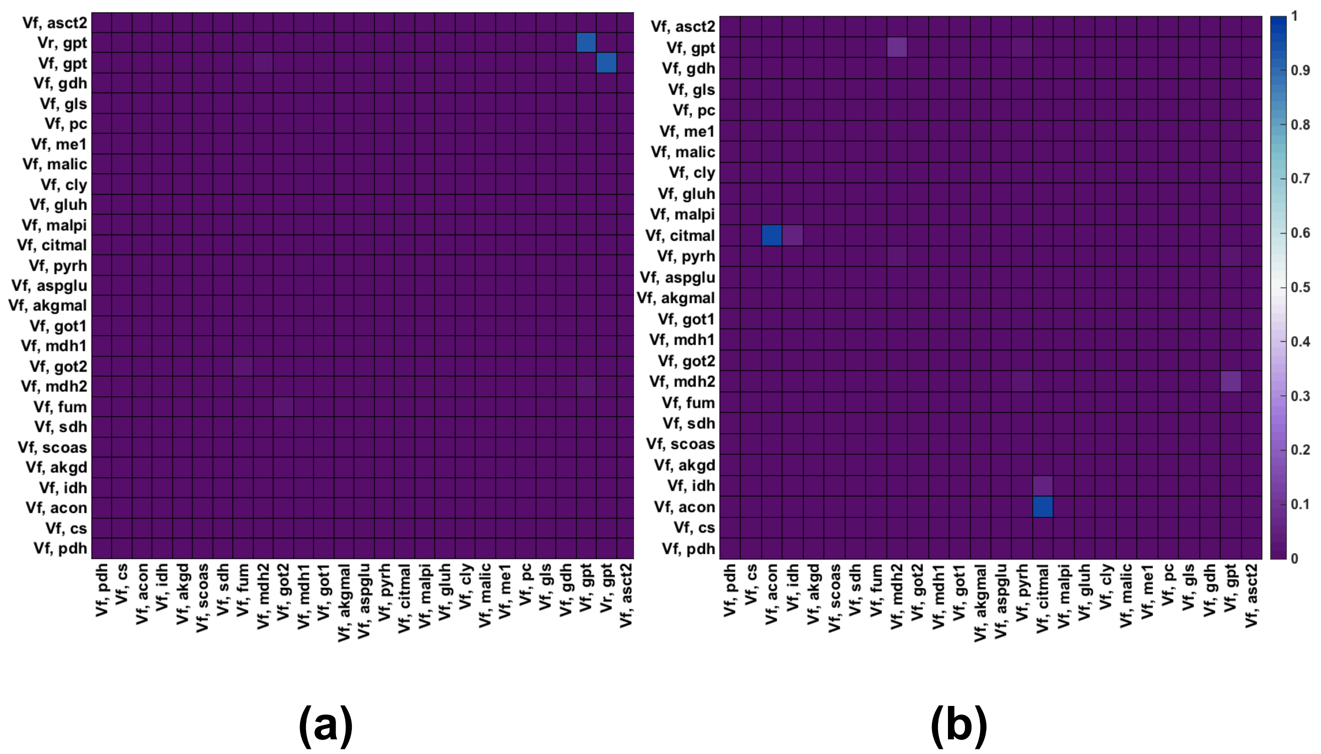

Figure S4. Results from the parameter identifiability analysis for reaction velocities involved in the TCA cycle Estimated correlation coefficients for **(a)** Round 1 and **(b)** Round 2. This analysis identified one set of correlated parameters in Round 1, enabling us to reduce the number of fitted reaction velocities from 27 to 26. The forward and reverse rate of the GPT reaction was found to be correlated.

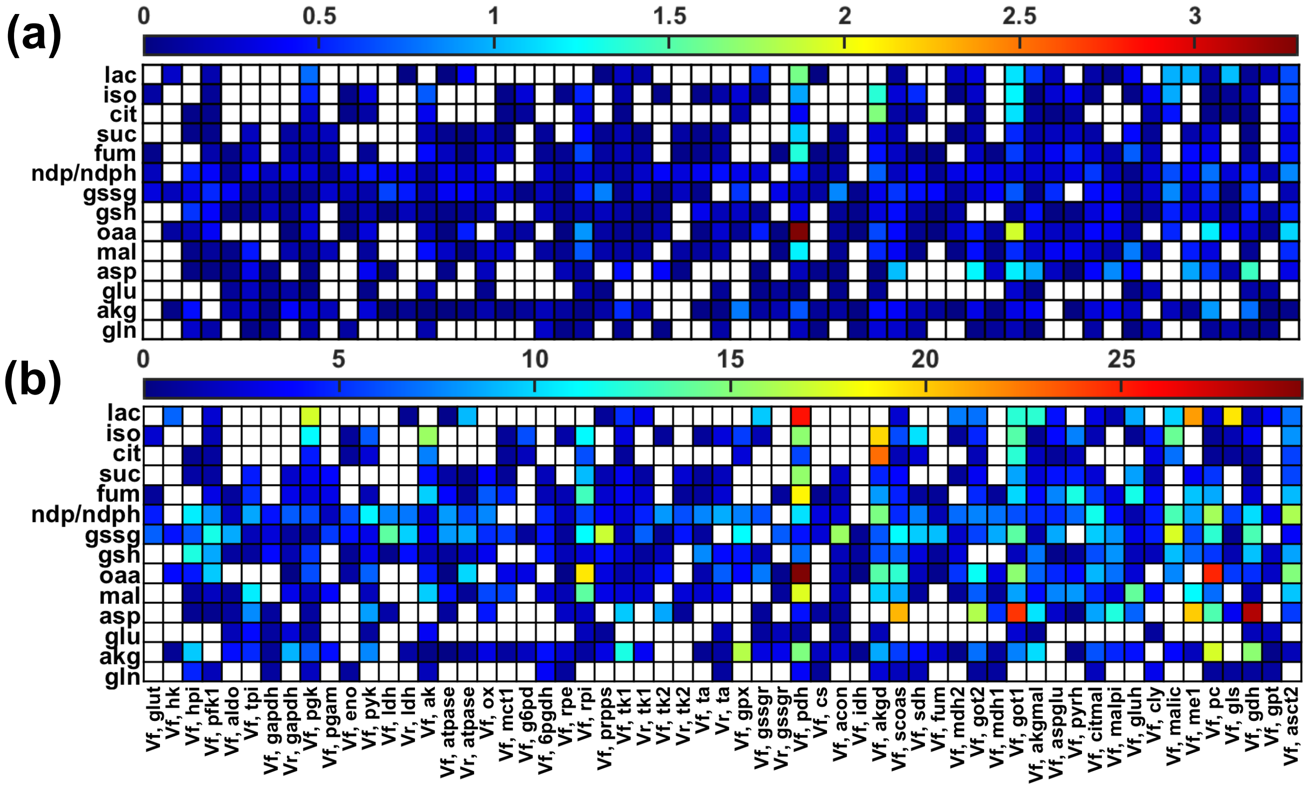

Figure S5. Results from the global sensitivity analysis. We performed a global sensitivity analysis, using the eFAST method, to determine how sensitive the fold-changes in the metabolite concentrations are to variance in the 59 reaction velocities. We sum the calculated sensitivity indices when the model is run for each of the 50 sets of initial conditions. In doing so, we determine which reaction velocities are influential across the various sets of initial conditions. **(a)** Cumulative first order FAST indices, S_i; **(b)** Cumulative total FAST indices, S_{ti}.

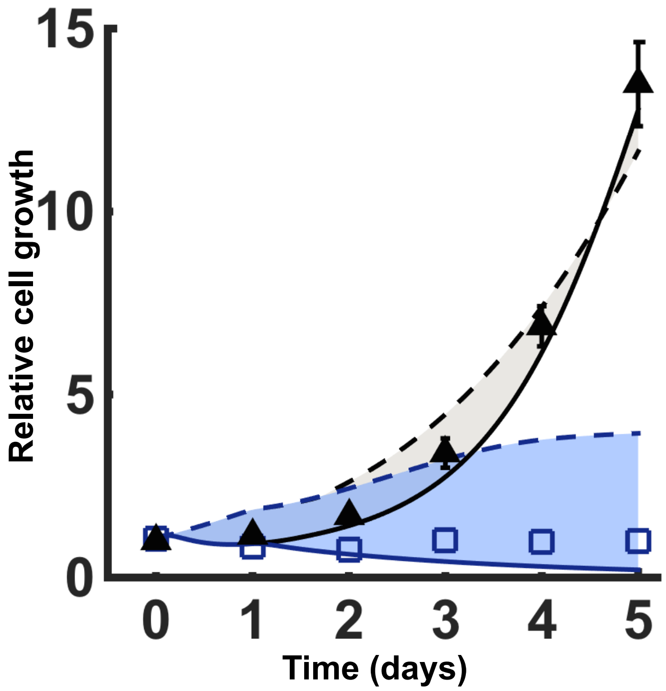

Figure S6. Results from model validation. Predicted relative cell count when cells are grown in complete media for five days (black) or in complete media for 24 hours, followed by glucose and glutamine deprivation for four days (blue). Triangles and squares represent the experimental data with error bars as available. The solid lines indicate the predicted result for IC #1, and the dotted lines indicate the predicted result for IC #2. The shading is used to draw the eye to the range of model predictions, based on the results form the two validated initial conditions.

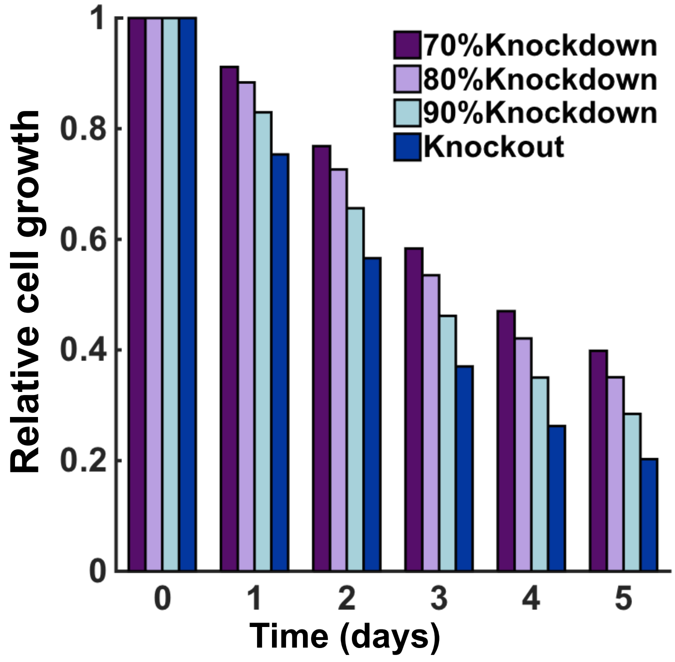

Figure S7. Results from varying percentage of GOT1 knockdown. Simulations for model fitting were conducted with 85% down-regulation of GOT1 enzyme activity. The cell growth is expected to be linearly affected with the percentage of knockdown as well as complete knockout. This is satisfied by model simulations under different knockdown percentage of GOT1 enzyme and a subsequent complete knockout.
